# “It’s Feasible to Write a Song”: A Feasibility Study Examining Group Therapeutic Songwriting for People Living With Dementia and Their Family Caregivers

**DOI:** 10.3389/fpsyg.2020.01951

**Published:** 2020-08-07

**Authors:** Imogen N. Clark, Phoebe A. Stretton-Smith, Felicity A. Baker, Young-Eun C. Lee, Jeanette Tamplin

**Affiliations:** Melbourne Conservatorium of Music, Faculty of Fine Arts and Music, University of Melbourne, Melbourne, VIC, Australia

**Keywords:** group therapeutic songwriting, music therapy, people with dementia, family caregiver, feasibility study

## Abstract

**Background:**

Psychosocial interventions for people with dementia and their family caregivers together may sustain relationship quality and social connection. No previous music therapy research has examined the effects of group therapeutic songwriting (TSW) attended by people with dementia/family caregiver dyads.

**Methods:**

This pre–post feasibility study aimed to examine the acceptability of a group TSW intervention for people with dementia/family caregiver dyads and test the sensitivity of the following outcomes: Quality of the Caregiver–Patient Relationship (QCPR, primary); Cornell Scale for Depression in Dementia (CSDD) and Quality of Life–Alzheimer’s Dementia for people with dementia, Patient Health Questionnaire-9, Assessment of Quality of Life–8 Dimensions (AQoL-8D); and Zarit Burden Interview for family caregivers. Six weekly 1 h sessions guided participants to identify preferred music, brainstorm ideas, create lyrics, and record songs. Qualitative interviews were conducted with dyads who completed the intervention.

**Results:**

Fourteen dyads were recruited and completed baseline assessments. Participants with dementia were aged 62–92 years (*M* = 77, SD = 11). Caregiver participants (11 spouses, two daughters, one son) were aged 54–92 years (*M* = 67, SD = 10.1). Four dyads withdrew owing to declining health or inconvenience before the program commenced (*n* = 2) and after attending 1–2 sessions (*n* = 2). Ten dyads formed four homogeneous TSW groups (71% completion). No statistically significant changes were detected for any measure. High QCPR ratings at baseline (*M* = 57.1) and follow-up (*M* = 57.4) demonstrated sustained relationship quality. For participants with dementia, large effect sizes for the CSDD suggested trends toward decreased depression (*d* = -0.83) and improved mood (*d* = -0.88). For family caregivers, a large effect size suggested a trend toward improvement for the AQoL-8D sub-domain examining independent living (*d* = -0.93). Qualitative data indicated that session design and delivery were acceptable, and TSW was a positive shared experience with personal benefits, which supported rather than changed relationship quality.

**Conclusion:**

High retention and qualitative data indicate that TSW was well received by participants. Effect sizes suggest that group TSW for dyads may have beneficial impacts on depression for people with dementia and quality of life for family caregivers. Future research with a fully powered sample is recommended to further examine the psychosocial impacts of group TSW for people living with dementia/family caregiver dyads.

## Introduction

Dementia is recognized as an international public health priority (WHO, 2017). Currently, approximately 50 million people live with a diagnosis of dementia worldwide, making it one of the leading causes of disability among older adults (Guerchet et al., 2016; WHO, 2017). Dementia is an umbrella term for a syndrome caused by several progressive neurological conditions, including Alzheimer’s disease, vascular dementia, dementia with Lewy bodies, and frontotemporal dementia (Arvanitakis and Bennett, 2019). A diagnosis of dementia and associated symptoms, including cognitive decline and changed behaviors, can have significant physical, psychological, and social impacts for the individual, as well as for spouses, other family members, and friends providing informal care (herein referred to as family caregivers). For example, people with dementia may experience changes related to cognitive processing, communication, self-care, mental health, and social connection, which in turn increase the likelihood of depression and anxiety among family caregivers as they manage the needs and autonomy of their family member while also attempting to maintain personal identity (Gorska et al., 2018). Accordingly, the World Health Organization emphasizes the importance of interventions and strategies that promote well-being and quality of life for both people with dementia and their family caregivers (Greenblat, 2012; WHO, 2017).

Many people with dementia and their family caregivers (herein referred to as dyads) experience a relationship strain as they navigate changing roles and responsibilities (Van Bruggen et al., 2016). Dyad relationships are further challenged as opportunities for reciprocity, communication, and shared meaningful experiences decline with the progression of dementia and/or when the caregiver experiences significant depression and stress (Ablitt et al., 2009). In addition, lack of understanding about dementia among family, friends, and the general public can restrict access to pleasurable and meaningful social activities as dyads avoid situations where they might be negatively judged (Batsch and Mittelman, 2012). In fact, a survey of 2,704 informal caregivers of people with dementia in the Netherlands identified restricted access to shared social activities as the most significant problem impacting relationship quality (Van Bruggen et al., 2016). However, it should also be noted that a strong mutual relationship between a person with dementia and their family caregiver is thought to enhance coping and sustain independent living through the progressive and unpredictable trajectory of neurodegeneration (Ablitt et al., 2009; Groen-van de Ven et al., 2017). Many dyads have explained how they continue to experience a mutually empowering relationship with interconnectedness, partnership, and closeness (Hellström et al., 2007).

Psychosocial opportunities that encourage reciprocity, meaningful interaction, and mutual support for people with dementia and their family caregivers together may have a positive impact on relationship quality, quality of life, and well-being for both (Ablitt et al., 2009; Van Bruggen et al., 2016; Wadham et al., 2016). Spousal couples, in particular, are likely to benefit from opportunities to attend mutually meaningful psychosocial activities owing to a shared identity and empathic attunement involving cherished memories and associations over long periods of time (Wadham et al., 2016). A recent systematic review conducted by Rausch et al. (2017) identified only seven studies that examined psychosocial programs attended by community-dwelling dyads together, with a range from skills-based to creative focus, and concluded that more good quality research is required to investigate the effectiveness of these interventions. Further, reviewers noted that a majority of programs were attended separately by either informal caregivers (*n* = 147) or people with dementia (*n* = 43), and no studies investigated psychosocial interventions with dyads where the person with dementia was living in residential care (Rausch et al., 2017). Evidently, there is a strong need for meaningful psychosocial interventions in both community and residential care contexts that are tailored for dyads comprising people with dementia and their family caregivers attending together (Van Bruggen et al., 2016; Rausch et al., 2017).

### Music-Based Psychosocial Interventions for People With Dementia/Family Caregiver Dyads

Music-based psychosocial groups provide engaging, socially stimulating opportunities that people living with dementia and their family caregivers can enjoy together (McDermott et al., 2014; Osman et al., 2016; Unadkat et al., 2017; Clark et al., 2018; Tamplin et al., 2018). To date, the majority of research has focused on community group singing programs with results suggesting that dyads attending these different programs experienced enhanced inclusiveness, social connection, confidence, coping, and growth (Camic et al., 2013; Osman et al., 2016; Unadkat et al., 2017). Research examining therapeutic group singing programs facilitated by qualified music therapists has also received recent attention (McDermott et al., 2014; Tamplin et al., 2018). For example, Tamplin et al. (2018) developed the Remini–Sing program to examine the effectiveness of a 20-weeks community-based therapeutic singing group for people with dementia and their family caregivers (*n* = 12 dyads) on relationship quality. In this feasibility study, the vast majority of dyads shared spousal relationships, though dementia severity and musical history of participants varied widely. While there were no significant differences in pre–post relationship quality or secondary measures examining health and well-being, qualitative analysis of interviews strongly suggested that the program led to many experiences of mutuality for dyads both within and beyond the group context (Clark et al., 2018; Tamplin et al., 2018). These findings suggest that music therapy interventions designed to meet the diverse needs of dyads have the potential to support relationship quality, as well as quality of life and well-being for both across the disease trajectory (McDermott et al., 2013).

### Group Therapeutic Songwriting With People With Dementia and Family Caregivers

Group therapeutic songwriting (TSW) is another applied music therapy intervention that could positively impact psychosocial outcomes for people with dementia and their family caregivers (Tamplin and Clark, 2019). Based on narrative approaches, TSW is a process that encourages participants to explore their thoughts, feelings, attitudes, and memories and guides them to translate ideas into lyrics and the creation of a song (Baker, 2015). Narrative therapy is recommended for people with dementia and their caregivers as a means for reflecting on life history, identifying strengths, and developing an understanding of their current situation (Wadham et al., 2016). An advantage of TSW over traditional counseling approaches and support groups is the gradual creation of a song over time, which requires prolonged engagement and mental and creative stimulation (Baker, 2015). When undertaken in a group setting with people in a similar situation, this process offers opportunities to work through challenges, share experiences, and express voice both within and beyond the group in meaningful and powerful ways (Baker and Stretton-Smith, 2017; Baker and Yeates, 2017; Baker et al., 2018).

While no research to date appears to have examined TSW for people with dementia/family caregiver dyads, music therapy research has investigated group TSW separately attended by either family caregivers of people with dementia (Baker and Yeates, 2017; Baker et al., 2018; García-Valverde et al., 2019) or people with dementia (Silber and Hes, 1995; Hong and Choi, 2011; Ahessy, 2017; Baker and Stretton-Smith, 2017). In a recent exploratory study, García-Valverde et al. (2019) found that caregivers who attended a 12-session group TSW program experienced reduced self-rated anxiety and depression and improved self-esteem and quality of life. These results confirmed an earlier feasibility study by Baker et al. (2018) demonstrating a moderate effect size for depression following a 6-weeks TSW group, suggesting that this variable may be sensitive to change. Analysis of qualitative interview data further suggested that TSW helped family caregivers build social connections, contextualize their experiences, and gain clarity around the carer journey, leading to personal growth and resilience (Baker and Yeates, 2017; Baker et al., 2018). Importantly, qualitative data have suggested that experiences of group TSW exceeded expectations (Baker and Yeates, 2017) and filled a gap not met by other carer support groups (Baker et al., 2018). These findings emphasize the potential value of TSW in supporting health, well-being, and coping in the carer role in creative ways that differ from traditional interventions and strategies, such as counseling and support groups.

Existing literature on TSW with people with dementia living in community and residential aged care contexts also indicates several benefits (Silber and Hes, 1995; Hong and Choi, 2011; Ahessy, 2017; Baker and Stretton-Smith, 2017). For example, a randomized controlled trial by Hong and Choi (2011) demonstrated improvement in participants’ global cognition, language function, orientation, and memory following a 16 weeks group TSW intervention utilizing lyric substitution and improvised song creation when compared to standard care alone. These outcomes are supported by qualitative interview data and clinical observations, which have indicated that TSW stimulates active engagement, learning, cognition, and language use (Baker and Stretton-Smith, 2017), as well as reminiscence and preserved song lyric and melodic memory (Silber and Hes, 1995; Ahessy, 2017). Baker and Stretton-Smith (2017) analyzed interviews with community-dwelling participants with dementia and day center care staff following a 10 weeks group TSW program and further found TSW to be a new and rewarding experience that, while including some challenges, enhanced connection and group cohesion and led to a sense of accomplishment and pride. This preliminary research highlights the capacity of people with dementia, suggesting that the stimulating and challenging aspects of TSW may be particularly beneficial. Qualitative findings further indicate potential benefits of TSW on the psychosocial well-being and quality of life of people with dementia, however, this has yet to be examined through quantitative outcome measures.

Despite the impacts of dementia spanning individuals and their families, there is limited existing research investigating the effects of psychosocial interventions for people with dementia and their caregivers together (Wadham et al., 2016; Rausch et al., 2017). While dyad-based group music therapy programs demonstrate promise, to date, this research has focused on the effects of therapeutic singing (Clark et al., 2018; Tamplin et al., 2018). To our knowledge, no previous studies have examined the effects of TSW on relationship quality between people with dementia and their family caregivers who attend groups together. Therefore, the current study aimed to examine the feasibility of a dyad-based group TSW program. The study also aimed to test the sensitivity and appropriateness of the primary outcome measure examining relationship quality and secondary outcome measures examining quality of life and depression (person with dementia and caregivers) and perceptions of the caregiver experience (family caregivers). A further aim was to analyze data from post-intervention interviews with each dyad to explore participant experiences of the group TSW program and intervention design.

## Materials and Methods

### Research Design

A single-group quasi-experimental pre–post design was used to examine the feasibility of a 6 weeks group TSW program. The study was funded by the [Dementia Australia Research Foundation – Hazel Hawke Research Grant]. Ethics approval was provided by [University of Melbourne] Human Research Ethics Committee (Ethics I.D 1851252.1). All participants either signed a written informed consent, or, if participants with dementia were unable to provide consent, a person responsible with power of attorney (not their family caregiver also participating in the study) provided consent. The study design was registered with the [Australian New Zealand Clinical Trials Registry, I.D ACTRN12618000919213).

### Participants

Participants voluntarily registered interest after receiving information through web pages, flyers, and verbal communications from various community services. Inclusion criteria for participants with dementia were (a) a diagnosis or probable or possible dementia and (b) fluent English language skills (either current or previously if nonverbal). Eligibility criteria for family caregivers were (a) aged 18 years or older and (b) fluent English language skills. Both participants with dementia and family caregivers needed to have functional hearing (with or without aids). As a feasibility study, we sought diverse participation and there were no restrictions based on living arrangement, relationship type (e.g., spousal, parent/adult child, other relative, friend), or the person with dementia’s diagnosis or severity of symptoms.

### Group Therapeutic Songwriting Intervention

Participants attended six 1 h weekly group TSW sessions in either community aged care support facilities or residential aged care homes. Homogeneous groups were formed based on relationship type (spousal or family) and living situations (living together in the community or separately because the person with dementia resided in a care home). Each group was facilitated by a qualified music therapist with an accredited degree and evidence of ongoing professional development.

A group approach was used based on positive findings from previous music therapy research including meaningful individual, shared, and social experiences from group interventions where dyads participated together (Unadkat et al., 2017; Clark et al., 2018; Tamplin et al., 2018), as well as from systematic reviews which suggest that these benefits may enhance feelings of mutuality (Wadham et al., 2016; Rausch et al., 2017). A protocol was developed to guide facilitation of the TSW sessions and meet the diverse needs of each participant as an individual and the group as a whole. This protocol drew on concepts of personhood (Kitwood and Bredin, 1992), couplehood (Hellström et al., 2005), and group process (Yalom and Leszcz, 2005) in combination with an experience-based TSW model (Baker, 2015). Taking these approaches into account, facilitation of TSW groups was driven by participants’ abilities and was adaptable to meet the strengths and resources of all group members from those with advanced dementia to family caregivers. Equipment included a whiteboard and markers, visual cues (e.g. photographs), devices for streaming commercial music, guitar, percussion instruments, lyric song books created by the therapist, and recording equipment for the final session. Session plans evolved over the 6 weeks intervention – from introductions and rapport building in session 1 to group song creations in sessions 2–5 and recording in session 6. Participants were also encouraged to listen to preferred music between sessions, identify favorite songs and key aspects that made them enjoyable (e.g., lyric lines, melody sections, instrumentation), and note down any original lyrical and musical ideas. Sessions, tailored to meet the needs of all participants in groups, included opportunities for brainstorming, reminiscence, and lyric creation as well as music listening, familiar song singing, and instrument playing.

### Assessment and Outcome Measures

The music therapists who facilitated groups administered assessments at baseline (1–4 weeks prior to the first TSW session) and post intervention (within 2 weeks of the final TSW session). The music therapists received training and were experienced in the administration of all outcome measures. Assessments were conducted at a convenient time for participants in their place of residence. Demographic information collected from the dyads recorded their age, gender, and relationship type. The Mini Mental State Examination (MMSE) was administered at baseline to gain a profile of cognitive function among participants with dementia (Folstein et al., 1975). The MMSE, an 11-item validated test of cognitive function for people with dementia, indicates range in cognitive impairment from normal (25–30) to mild (20–24), moderate (13–20), and severe (less than 12).

The primary outcome measure (dyad relationship quality) was examined using the Quality of the Caregiver–Patient Relationship (QCPR) (Spruytte et al., 2002). The QCPR is a 14-item self-report scale completed by family caregiver participants using a 5-point scale (totally disagree to totally agree). Total QCPR scores are derived from two dimensions measuring levels of criticism (range of 6–30) and warmth (range of 8–40). Total scores range from 14 to 70, with higher scores reflecting increasing relationship quality. Spruytte et al. (2002) suggest that a strong relationship is indicated by scores > 42. The QCPR has demonstrated acceptable internal consistency (*α* = 0.82) and concurrent validity and has been used in other trials to examine the effects of music therapy interventions on relationship quality between people with dementia and their family caregivers (Baker et al., 2018; Tamplin et al., 2018).

Participants with dementia were assessed using the Cornell Scale for Depression in Dementia (CSDD) and the Quality of Life–Alzheimer’s Dementia (QoL-AD). The CSDD is a 19-item interviewer-administered evaluation that derives information from both the person with dementia and his or her caregiver (family or formal) through two separate semi-structured interviews (Alexopoulos et al., 1988). Items on the CSDD are rated and summed across domains measuring well-being, sleep, appetite, and symptoms of dementia for the preceding week on a scale from absent or nonassessable (0), mild or intermittent (1), to severe (2). Total scores indicate: no depressive symptoms (0–5), possible depression (6–9), probable major depression (10–17), and definite major depression (18–38). The CSDD demonstrates acceptable reliability and validity for use in trials (Alexopoulos et al., 1988) and is the recommended instrument of choice for trials examining depression and mood in people with dementia (Moniz-Cook et al., 2008).

The QoL-AD is a 13-item interviewer-administered questionnaire examining quality of life for people with cognitive impairments (Logsdon et al., 1999, 2002). Quality of life can be assessed separately from the perspective of the person with dementia (self) and the family/formal caregiver (proxy). Items assessing mental health and social and financial domains are rated using a 4-point Likert scale ranging from poor (1) to excellent (4) and summed to yield a score ranging from 13 to 52. Higher scores indicate increased quality of life. The QoL-AD has demonstrated acceptable reliability and validity and is recommended as the measure of choice for studies examining quality of life in older adults with cognitive impairments (Logsdon et al., 1999; Moniz-Cook et al., 2008).

Family caregiver participants were assessed using the Assessment of Quality of Life–8 Dimensions (AQoL-8D), Patient Health Questionnaire-9 for Depression (PHQ-9), and Zarit Burden Interview (ZBI). The AQoL-8D is a 35-item self-rated evaluation of quality of life over the preceding week (Maxwell et al., 2016). Of the eight dimensions, three are related to the physical super-dimension (independent living, senses, pain) and the other five relate to the psychosocial super-dimension (mental health, relationships, coping, self-worth, happiness) (Richardson et al., 2014a). Improvement is indicated by a reduction in ratings globally and for each dimension. For the purposes of the current project, unweighted AQoL-8D responses were entered into an online scoring algorithm to calculate psychometric scores examining health-related quality of life (Richardson et al., 2014a).

The PHQ-9 is a nine-item self-rating module for measuring symptoms of depression from the full Patient Health Questionnaire (Kroenke et al., 2001). Items identifying symptoms of depression are rated for occurrence over the previous 2 weeks from not at all (0), several days (1), more than half the days (2), and nearly every day (3), with total scores ranging from 0 to 27. Summed scores denote the following categories of severity: no depression (0–4), mild depression (5–9), moderate depression (10–14), moderately severe depression (15–19), and severe depression (20–27). The PHQ-9 demonstrates acceptable sensitivity and specificity across diverse patient samples (Kroenke et al., 2001).

The ZBI is a 22-item measure examining perceived carer burden (Zarit et al., 1980). Carers are asked to select a response that best describes how they feel about aspects of caring from never (0), rarely (1), sometimes (2), quite frequently (3), and nearly always (4). Responses for each item are summed, and levels of perceived burden are interpreted as none to a little (0–20), mild to moderate (21–40), moderate to severe (41–60), and severe (61–88). The ZBI demonstrates acceptable reliability and validity for examining burden experienced by informal family carers of people with dementia (Hebert et al., 2000; Seng et al., 2010).

### Post-intervention Interviews

Audio-recorded qualitative interviews were conducted with each dyad during the post assessment. Interviews were conducted by the same music therapist who facilitated the dyad’s TSW sessions. Interview questions focused on how dyads experienced participating in TSW, including their experience of working as a group and attending with their spouse/family member, as well as what stood out or was meaningful (if anything). Dyads were also asked to comment on the acceptability of the intervention and session design (e.g., session length, number, and frequency).

### Data Analysis

Session attendance and participant demographics (e.g., age, gender, dyad relationship and living situation, musical background, and cultural backgrounds) were recorded descriptively, and, where appropriate, means and standard deviation were calculated. MMSE mean scores and standard deviation were calculated to provide a profile of cognitive function among participants with dementia at baseline.

Primary and secondary outcome measure data were analyzed using paired sample *t*-tests with a significance level of 0.05. We also applied the Shapiro–Wilks test to all outcomes and performed the equivalent nonparametric Wilcoxon signed rank test when abnormally distributed data were observed. As this was a feasibility study with a small sample size, statistical significance was not expected. Rather, effect size calculations on pre to post changes for all outcome measures were calculated to examine clinical relevance and sensitivity to change (small *d* = 0.2; medium *d* = 0.5; large *d* = 0.8) (Cohen, 1988).

We planned to impute missing data according to the relevant recommendations for each outcome measure. For the QCPR, PHQ-9, and QoL-AD, if less than 20% of the total available items were missing, we imputed the mean value of scored items, and if 20% or more items were missing, the individual’s score was excluded from analysis (Fox-Wasylyshyn and El-Masri, 2005). For the Zarit Burden Interview (ZBI), data were imputed using the median scale width when less than 20% of items were missing, and if 20% or more items were missing, the individual’s score was excluded from analysis (Zarit et al., 1980). For the CSDD, nonassessable items were imputed with the lowest possible score, zero (Wongpakaran et al., 2013). The AQoL-8D utility algorithm (unweighted) was used to impute values for missing data within each dimension (Richardson et al., 2014a, b; Maxwell et al., 2016). AoL-8D dimensions with 3–4 items allowed for one missing value to be imputed, dimensions with 7–8 items allowed for two missing values, and when more responses were missing, the individual’s score was excluded from analysis of that dimension.

Qualitative data were analyzed using interpretative phenomenological analysis, as informed by Smith et al. (2009). A more in-depth description of qualitative interview data and analysis is planned for another paper by the same authors. However, in keeping with the purpose of this paper, interview data were examined in relation to the feasibility of the TSW intervention. This included how participants described their experiences of group TSW sessions, focusing on practical aspects of session delivery, the group context and experience of attending together as dyads, as well as perceived barriers and enablers to participation.

## Results

**FIGURE 1:**
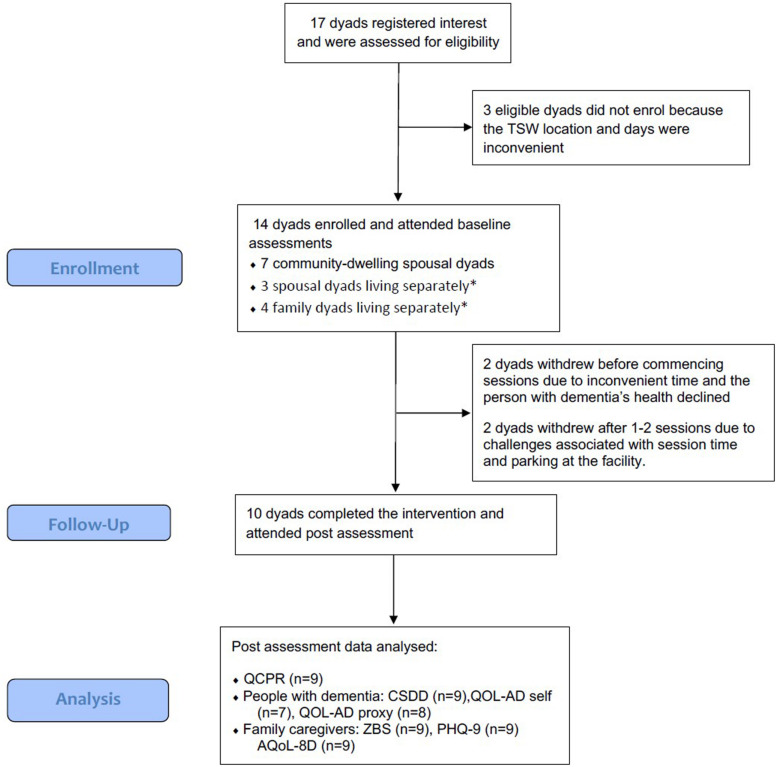
Participant Flow (^∗^participant with dementia living in residential care).

Participants were recruited between April and October 2018. Of the 17 dyads who registered interest in the project, 14 consented to participate and completed baseline assessments. Time since diagnosis for participants with dementia ranged from 1 to 9 years prior to the study, and cognitive function based on MMSE scores ranged from normal (*n* = 1) to mild (*n* = 2), moderate (*n* = 5), and severe (*n* = 6) (Table 1, Baseline characteristics of participants). In total, 10 of the 14 enrolled dyads completed the TSW program and participated in post assessments (71% completion) (Figure 1, Participant Flow).

**TABLE 1:** Participant characteristics at baseline.

	Participants with dementia			Family caregivers		
	Mean	*SD*	Range	Mean	*SD*	Range
Female (male)	7 (7)	–	–	9 (5)	–	–
Age (years)	77	11	62–92	67.1	10.1	54–92
MMSE/30	12.5	9	0–29	–	–	–

*MMSE, Mini Mental State Examination.*

The 10 dyads who completed the TSW program were allocated into four homogeneous groups with 2–3 dyads (4–6 participants) per group. Two groups involved community-dwelling spousal couples, and the other two groups involved dyads living separately where the person with dementia resided in a care home (one spousal and one family group). Most participants were born in Australia (*n* = 15), and others were born in Lebanon (*n* = 2), Italy (*n* = 2), and Malaysia (*n* = 1) (Table 2, Group allocations and participant background).

**TABLE 2:**
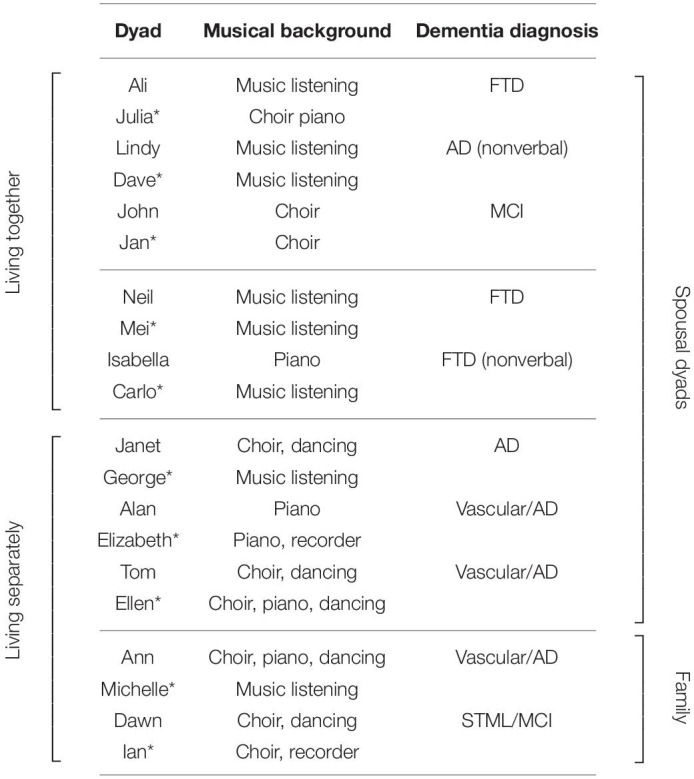
Participants background and group allocations.

All names are pseudonyms; *family caregiver. FTD, frontal temporal lobe dementia, AD, Alzheimer’s dementia; MCI, mild cognitive impairment; STML, short-term memory loss.

The six TSW sessions were held from early October 2018 until mid-January 2019 with a 1- to 2-week break over the end-of-year period. Average attendance of the six available sessions was 5.3 across all participants (88%), 5.7 for participants with dementia (95%), and 5.1 for caregiver participants (85%). One dyad from a community spousal group missed two sessions as the caregiver was unwell and the person with dementia was in respite care. Three caregivers from residential groups (one spousal and two family) missed one to two sessions because of other commitments, but their family member with dementia still attended.

Groups wrote and recorded 2–3 songs over the 6 weeks (10 songs in total across the four groups). All groups chose to use the song parody method for at least one song, which involved rewriting original lyrics to familiar and preferred tunes. One community spousal group also chose to compose a song using song collage (combining two familiar melodies with original lyrics) and by setting original spoken word poetry to background music.

### Quantitative Results

Aside from withdrawals (*n* = 4), substantial amounts of missing data for participants with dementia meant that the following cases were excluded from outcome measure analyses: CSDD *n* = 2 pretest, *n* = 1 posttest; QoL-AD self-rated *n* = 3 pretest, *n* = 4 posttest; QoL-AD proxy-rated *n* = 2 pretest, *n* = 3 posttest. Four participants with dementia found it too difficult to maintain focus required to complete the self-rated QoL-AD, and of these, three had an MMSE score less than 10. Missing data also occurred when caregiver participants either did not feel confident about accurately completing measures as a proxy or felt that their spouse/family member could complete self-report measures for themselves.

Apart from withdrawals (*n* = 4), missing data from caregiver participant assessments were minimal. However, post data were not collected for any outcome measures for one family caregiver who had completed baseline assessments as five of the six TSW sessions were attended by her sister, rather than herself. One other family caregiver participant refused to rate an item examining suicidal ideation on the depression scale.

There was no statistically significant pre to post change for the primary outcome measure, the QCPR examining relationship quality. Pre and post total QCPR scores demonstrated high relationship quality at both time points. Similarly, pre to post results for the QCPR subscales demonstrated high levels of warmth and low levels of criticism at both time points (Table 3, Relationship quality).

**TABLE 3:** Relationship quality (primary outcome).

QCPR	Pretest (*SD*) *N* = 14	Posttest (*SD*) *N* = 9	Pre–Post Diff (*SD*) 95% CI
Total	57.1 (4.8)	57.4 (3.7)	−0.4 (5.7) −4.8 to 4.0
• Warmth	33.6 (3.1)	34.1 (3.3)	−0.55 (4.8) −4.3 to 3.2
• Criticism	23.5 (2.8)	23.3 (2.7)	0.19 (2.1) −1.4 to 1.8

*QCPR, Quality of the Caregiver–Patient Relationship.*

There were no statistically significant pre to post differences for secondary outcomes examining participants with dementia depression (CSDD) and quality of life (QoL-AD self-rated and proxy). CSDD ratings indicated probable major depression at pretest (*M* = 11) and possible depression at posttest (*M* = 7.9), and a large pre–post effect size for the total score was observed for positive changes in depressive symptoms (*d* = -0.82, CI = -1.7 to 0.1). Small to large pre–post effect sizes for CSDD subscales were observed for positive changes in mood (*d* = -0.88, CI = -1.7–0.6), behavioral disturbance (*d* = -0.74, CI = -1.6 to 0.2), cyclic functions (*d* = -0.33, CI = -1.2 to 0.6), and ideational disturbance (*d* = -0.59, CI = -1.5 to 0.3). Small to moderate effect sizes on the QoL-AD were observed for negative changes in self-rated (*d* = -0.66, CI = -1.7 to 0.4) and proxy-rated (*d* = -0.36, CI = -1.3 to 0.6) quality of life (Table 4, Results for participants with dementia).

**TABLE 4:** Results for participants with dementia (secondary outcomes).

Outcome (range)	Pretest (*SD*)	Posttest (*SD*)	Pre–Post Diff (*SD*) 95% CI

CSDD	*N* = 12	*N* = 9	
Total	11.0 (3.9)	7.9 (3.6)	3.1^+^ (5.7) −1.7 to 7.9
• Mood	4.4 (1.9)	2.9 (1.4)	1.5^+^ (2.4) −0.5 to 3.5
• Behavior	2.4 (0.9)	1.6 (1.3)	0.8^#^ (1.3) −0.3 to 1.8
• Physical	1.0 (0.5)	1.1 (1.3)	−0.1 (1.4) −1.3 to 1.0
• Cyclic	1.3 (1.5)	0.9 (0.6)	0.4* (1.8) −1.1 to 1.9
• Ideational disturbance	1.5 (2.1)	0.5 (0.9)	1.0^#^ (1.6) −.3 to 1.8

**QoL-AD**	**Self-rated *N* = 11 Proxy-rated *N* = 12**	**Self-rated *N* = 6 Proxy-rated *N* = 7**	

• Self-rated	39.6 (4.5)	36.8 (3.8)	2.8^#^ (3.6) −1.7 to 7.3
• Proxy	31.3 (3.6)	30.0 (3.5)	1.3* (2.3) −1.0 to 3.7

CSDD, Cornell Scale for Depression in Dementia; QoL-AD, Quality of Life-Alzheimer’s Dementia; ^+^large effect size; ^#^medium effect size; *small effect size.

There were no statistically significant pre to post differences for secondary outcomes examining caregiver quality of life (AQoL-8D), depression (PHQ-9), or perceived burden of care (ZBI). A large pre–post effect size was observed for positive changes in the AQoL-8D independent living subscale (*d* = -0.93, CI = -1.8 to 0), and small effect sizes were observed for positive changes on the physical super-dimension (*d* = -0.48, CI = -1.3 to 0.4), happiness subscale (*d* = -0.42, CI = -1.3 to 0.4), and relationships subscale (*d* = -0.32, CI = -1.2 to 0.5). There was also a small pre–post effect size observed for negative changes on the AQoL-8D coping subscale (*d* = 0.24, CI = -0.6 to 1.1). Caregiver burden was rated as mild to moderate at both time points, and small effect sizes were observed for decreases in perceived burden (*d* = -0.45, CI = -1.3 to 0.4) and depression (*d* = -0.20, CI = -1.0 to 0.6) (Table 5, Results for caregivers).

**TABLE 5:** Results for caregivers (secondary outcomes).

Outcome measure	Pretest (*SD*)	Posttest (*SD*)	Pre–Post Diff (*SD*) 95% CI

AQoL-8D	*N* = 14	*N* = 9	
Global	70.4 (10.1)	68.5 (11.5)	2.0 (7.4) −3.6 to 7.6
• Independent living	87.0 (9.2)	76.5 (14.1)	10.5^+^ (14.6) −6.9 to 21.7
• Happiness	62.5 (12.5)	56.3 (17.7)	6.3* (9.9) −1.3 to 13.9
• Mental health	64.0 (16.4)	65.0 (17.2)	−1.0 (9.9) −8.7 to 6.6
• Coping	60.2 (16.0)	63.9 (13.8)	−3.7* (11.9) −12.9 to 5.4
• Relationships	73.3 (10.1)	69.6 (13.5)	3.7* (16.4) −8.9 to 16.3
• Self-worth	63.0 (18.2)	63.0 (19.1)	.0 (15.6) −12.0 to 12.0
• Pain	74.4 (19.2)	77.8 (19.2)	−3.3 (14.1) −14.2 to 7.5
• Senses	81.2 (4.1)	81.2 (4.1)	.0 (3.9) −3.0 to 3.0
• Physical super-dimension	82.1 (7.0)	78.3 (9.3)	3.8* (8.6) −2.8 to 10.4
• Psychosocial super-dimension	65.7 (12.2)	64.4 (14.5)	1.2 (8.8) −5.6 to 8.0

**PHQ-9**	***N* = 14**	***N* = 9**	

Total/27	17.0 (6.6)	15.7 (6.0)	1.3* (3.4) −1.3 to 3.9

**ZBI**	***N* = 14**	***N* = 9**	

Total/80	33.7 (8.9)	30.1 (6.4)	3.5* (11.9) −5.6 to 12.7

ZBI, Zarit Burden Interview; PHQ-9, Patient Health Questionnaire-9 (depression); AQoL-8D, Assessment of Quality of Life–8 Dimensions; + large effect size; # medium effect size; *small effect size.

The Shapiro–Wilks test demonstrated abnormality for some outcomes. Therefore, we performed the equivalent nonparametric Wilcoxon signed rank test across all outcomes, which confirmed all results from parametric analyses.

### Qualitative Results

In-depth results of the full interpretative phenomenological analysis of interview data are planned for a separate manuscript. In this paper, qualitative data were used to examine the acceptability of the intervention, focusing on salient features of dyads’ described experiences of TSW, as well as participant perceptions of barriers and enablers, and aspects of session delivery and design.

#### Dyads’ Experiences of Group Therapeutic Songwriting

TSW was overwhelmingly recognized as a positive, worthwhile, and enjoyable experience that participants were willing to commit time to. Importantly, the vast majority of dyads commented on appreciating being able to attend together and highlighted the value of TSW as a new, creative, and stimulating shared experience.

*“Just in itself, yes [it was worthwhile], for sure. Yeah, yeah, yeah. Just in its own right, as a thing to do, will I commit the time to come and do this? Absolutely. Yeah, it was good.” (Ian^∗^)*^1^

“It’s more enjoyable coming with Mei […] I found it more enjoyable with the both of us.” (Neil) “Together. Yeah, it’s more enjoyable coming together.” (Mei^∗^)

“For the three couples that were there, it was something that we hadn’t done before […] I thought it was terrific from that point of view. That it got people, sort of, motivated and thinking.” (Dave^∗^)

Although all dyads described TSW as “new” and “different,” half of the dyads interviewed also commented that the experience of participating together felt “natural” (Michelle/Ann) and “normal” (Ellen/Tom), explaining that they had always had a “very strong bond” (Carlo/Isabella) and “done everything together” (Jan/John). In this sense, the TSW program appeared to largely support, rather than change, existing relationship quality.

“We don’t normally do that [laughing]. We don’t normally sing together, do we? […] So yeah, it was completely [different] […] [but] over the years, we’ve done lots of things together, so being together is quite natural.” (Michelle^∗^)

“I think we’re just used to being together (Ellen^∗^). We’ve done that many things, we’ve done (Tom). I go to activities with him quite often here […] So we’re sort of used to working together (Ellen^∗^). As I’ve said before, we’ve got on pretty well for 70-odd years.” (Tom)

Despite some family caregivers initially being incentivized to attend for their family member with dementia, all participants recognized personal benefits for both members of the dyad, including joy, motivation, pride, and achievement. Two participants in particular (one participant with dementia and one family caregiver) also recognized a positive impact on mood. Further, caregivers found meaning in witnessing their family member and others enjoying, gaining from, and contributing to the TSW process.

“First, I thought, well I’m here for Isabella’s benefit […] but after the first session I sort of loosened up […] I pushed myself to come [after being ill] because I wasn’t really feeling that well. But once I was here, it just snapped me out of it and I really enjoyed it.” (Carlo^∗^)

“It was nice [for me] to reminisce, as well. But you don’t always have a lot of things that make you happy. So, it was nice to see you enthusiastic about doing something and coming back feeling good in yourself (Michelle^∗^). Yeah, well I agree with that (Ann) […] The look on Mum’s face and the look on Dawn’s face just said it all.” (Michelle^∗^)

Participants also focused on TSW as a positive social opportunity that was different to other group experiences. Dyads identified the goal and process of TSW and use of music as important factors prompting interaction, collaboration, and connection.

“The context of what you were trying to achieve, the one goal […] the music allowed you to sort of interchange with other people. […] I thought that was one of the big pluses.” (Dave^∗^)

“Here I find this more interactive [than other group experiences] […] with the music, you have to interact […] with other people. Even with each other, too.” (Mei^∗^)

#### Enablers, Barriers, and Types of Participation in Therapeutic Songwriting

All dyads described how facilitation methods supported the achievability and success of TSW, including for people who had never written a song before. Dyads were surprised by “the ease of the whole process” (Carlo^∗^), largely attributing this to supportive aspects of facilitation and therapeutic “leadership,” including the therapist prompting and encouraging participants to “have a go” (Michelle^∗^) and suggesting and organizing the groups’ thoughts and ideas.

“I thought it would be difficult. But the way it’s done, it makes it quite easy for us to compose the songs […] Before we started, I thought how could we ever write songs? […] I was just worried about the composing because I have no musical background.” (Mei^∗^)

“[The music therapist] made it a lot easier, you know, by prompting us and coming up with thoughts and things for us to start us off […] It made a big difference.” (Carlo^∗^)

“[The music therapist’s] lead of getting all those thoughts up on the board, that was important, that was really good.” (Ian^∗^)

Dyads recognized that participants with dementia engaged in the TSW process at varying levels – from “on the day” in the here-and-now to self-directed lyric writing outside of sessions. Participants also identified aspects of the approach and facilitation that were important in supporting and including all participants, especially those with more advanced dementia or who were nonverbal or used assistive communication devices. These included choosing the “right songs” (Ian^∗^), the therapist leaving “space” (Ali) for participants to respond and contribute, and integrating singing, instrument playing, music listening, and visual prompts into the TSW program.

“I thought it was interesting with John, that he was able to go away and think about it and put things together and come back. That so much wasn’t the case with Lindy […] I think Lindy still got a lot out of it on the day.” (Dave^∗^)

“Having the slower songs made it so much easier […] Mum’s got to read it and process it […] [so the] slower song just made it so much easier for them to be able to sing.” (Michelle^∗^)

As well as facilitation methods, participants recognized the role of family caregivers in supporting and “guiding” their family member with dementia. Participants commented that having both members of the dyad in the group was the “best way to do it” (Ian^∗^).

“Oh, I think like having myself and Michelle there helped to put the spark out, to refresh the memory, just to get those things rolling […] So it was important, I think, it was the best way to do it to have both of us there. Both generations.” (Ian^∗^)

“Ellen knows what she’s doing and knows the music. She guides me (Tom) I try to help you (Ellen^∗^) […] It’s like [the music therapist] guiding me.” (Tom)

While participants were able to identify many enablers, they also described barriers. One family caregiver commented that her husband experienced difficulties adjusting to the group setting, while another family caregiver noticed that his wife and some other participants with dementia had difficulty maintaining focus during TSW. Lyric writing was recognized as a valuable and gratifying experience for the majority. However, one participant who was in the more advanced stages of dementia and nonverbal was unable to contribute to lyric writing. Similarly, while the recording session was described by many as the “climax” (Ellen/Tom) of the program due to it being a new and intriguing experience, one family caregiver felt that the “new equipment [and] close proximity to people” (Carlo) presented challenges for his wife.

“The only thing I would say is, with people who are sort of fairly advanced with the illness, you know, the lyric writing probably isn’t beneficial for them. You know, for people like Neil it was fine. But people like Isabella, it just, yeah […] [the recording session was also difficult]. She wouldn’t know what was going on and what she was here for. And when she’s in new environments she gets a bit anxious and agitated.” (Carlo^∗^)

However, despite these challenges, all dyads recognized TSW as worthwhile.

“*Yeah, I think [TSW was worthwhile for Isabella], I think it was. Listening to [the music therapist] playing the guitar and singing and all that, she was very intrigued and very connected when that was happening.” (Carlo^∗^)*

#### Therapeutic Songwriting Intervention Design and Delivery

Overall, participants commented positively on the intervention design and delivery, including group size, frequency and duration of sessions, and TSW methods. Regarding group size, one participant wondered whether a slightly larger group would be helpful to compensate for weeks when people were unable to attend. However, generally, participants felt the groups of 2–3 dyads were enough to trigger thoughts and exchange ideas, while still being able to reach a consensus and value individual contributions. Further, participants speculated that the small group context may be easier than TSW with larger groups or individual dyads.

“With the smaller group, you could toss [ideas] around and you got the yes or no […] I think it’d be a lot harder if you tried to do it with just one couple. I think you needed that input from […] other people as to their experiences and ideas.” (Dave^∗^)

“It might have been harder to get consensus [in a larger group]. You know, with the more people, people start to head in different directions, and you get nothing done […] [in this group] you only had to say it and it was done!” (Ian^∗^)

Six weekly sessions were generally perceived as an appropriate amount of time for composing 2–3 songs using song parody and/or song collage techniques. However, some suggested that the program could have been extended. One family member explained it could have gone for longer due to the time taken for participants to adjust and “get into it” (Elizabeth^∗^). Others stated that they “didn’t like it stopping” (Ann) or that an ongoing program would “help dramatically” (Jan^∗^), even on a less regular basis. One participant expressed that TSW sessions could have been more frequent, such as “every two or three days” (George^∗^), although acknowledged this may be difficult for others.

“Yeah, [the 6 weeks] was good. It could have even gone a little bit longer, I think, maybe. As I said, it took a little bit for them to get into it” (Elizabeth^∗^).

“It’d be great if we have […] that sort of group going. Because in between whiles, you know, we could just sort of say, hang on, we’ve got to have something new for the group […] If that was there, you know, on a two-weekly, three-weekly, four-weekly basis, I reckon it would help dramatically.” (Jan^∗^)

“You could run it every two or three days. But I mean it’s not… it’s difficult for other people as well as yourself what who’s doing it.” (George^∗^)

One-hour sessions were experienced as “just right” (Neil/Mei). Sessions were long enough to develop and work on new ideas, but not too long as to be difficult for participants to sustain focus. At the same time, one dyad appreciated recommendations from the music therapist to arrive half an hour early to allow time for socialization. One dyad also suggested that sessions in the morning rather than late afternoon may be “better” (Elizabeth^∗^) in residential care as people with dementia might experience less confusion and find it easier to participate at that time.

“It’s hard to…you can’t have long really long sessions. They’ve got to be short sessions… because they just yeah, they can’t focus for a long time.” (George^∗^)

“[The] suggestion of coming that half hour early was wonderful because without that we wouldn’t have had any time at all. So yeah, that part is, encourage people to come that half-hour early and get the chatting out of the road.” (Jan^∗^)

Regarding TSW methods and techniques, the majority of participants recognized song parody as an appropriate level of challenge and considered original music creation as “too much to ask” (Ian^∗^). One participant with dementia also expressed enjoyment of the song collage technique, and others highlighted the value of reminiscence-focused TSW.

“[I enjoyed] the way you can change a song into another song […] the different melodies.” (Neil)

“If you’ve got to actually compose a bit of music, it would be [difficult]. We’ve sort of done it to other tunes that we know. It’d be hard to come up with a new tune though.” (Elizabeth^∗^)

“Well first we had to pick the songs, didn’t we? Which, there was a chance we were going to actually come up with our own songs, as well, but we quickly moved past that idea [laughing]. It was a bit too much to ask…But having a tune and some ideas and then putting them together, it was good fun […] [and] actually, the reminiscences that came up, that then, you know, went into the words, was really interesting.” (Ian^∗^)

“I think [it’s] better if, as we did, we talked about our previous life […] it brought the past out of people who are now sitting in this environment […] Because I think there’s a bit more happiness.” (George^∗^)

Finally, the majority of dyads described appreciating having an “end product” in the form of a recording, which enabled them to listen back to the final song creations, gain a sense of completion, and recognize the achievements of the group.

“At the end it sounded, um, I mean so much better than what I thought it was going to sound […] I think maybe the end product [is what stood out]! We wrote the song and then to actually, you know, hear it at the end.” (Elizabeth^∗^)

“Knowing that we’d completed it and, ah, hearing it, that was really the best part for me, I think. Knowing that we’d actually accomplished it.” (Ellen^∗^)

## Discussion

This feasibility study aimed to examine the acceptability of a 6-week group TSW program and research protocol, as well as the sensitivity of outcome measures examining dyad relationship quality, depression, and quality of life of participants with dementia and family caregivers and family caregivers’ perceptions of their carer role. High retention (71%) and session attendance (88%) rates coupled with positive experiences described in the qualitative interview data suggest that the TSW program was accessible and acceptable for dyads attending groups in both community and residential care contexts. Reasons for withdrawing before completing the follow-up assessment (*n* = 4), including inconvenient session time and declining health of the person with dementia, were understandable and expected based on other music therapy research with a similar population (Tamplin et al., 2018). Participants felt that the six weekly 1 h sessions were acceptable and at times suggested prolonging the intervention, signifying that the intervention period did not impose any undue burden on participants. Qualitative data also indicated that dyads were highly committed to the project, with only a few who missed sessions because of illness or unavoidable appointments. The adaptive and flexible approach to TSW used in the project may have supported dyads to reengage with the process after a period of absence and therefore met the needs of a population where health status and competing needs impact attendance.

Results suggest that our TSW protocol for dyads, incorporating person-centered, couple/family-centered and group process concepts and approaches, successfully engaged people with diverse needs attending the groups. Despite significant diversity in cognitive capacity as measured using the MMSE (*M* = 12.5, *SD* = 9.0, range = 0–29), participants explained how people with advanced dementia appeared to “enjoy” themselves “on the day” (in the here-and-now), while others benefited from the mental challenge both during and outside sessions. It is also worth noting that while the final recording session was a highlight for some people with dementia, others found this session challenging because of the newness and unfamiliarity of the situation. These findings recognize the importance of clinical judgment to gauge the response and support engagement of individuals with dementia over different phases during the program. Caregivers also explained how the sessions met their own psychosocial and mental health needs, with some reporting that TSW had a positive impact on mood. Our study findings support previous TSW research, indicating that TSW is a unique intervention that rekindles inner strength and stimulates personal growth for both people with dementia and family caregivers (Baker and Stretton-Smith, 2017; Baker et al., 2018). These qualitative findings shed light on our quantitative results, which suggest that group TSW with people with dementia/family caregiver dyads might improve important health and well-being outcomes for both members of the dyad.

The primary outcome measure, the QCPR, demonstrated that high relationship quality at baseline was sustained at follow-up. These results are consistent with research examining cognitive therapy (Cove et al., 2014) and music therapy (group therapeutic singing) for people with dementia and their spousal/family caregivers (Tamplin et al., 2018) following intervention periods up to 20 weeks, suggesting a ceiling effect and/or potential lack of sensitivity of QCPR measure to change over the short-term. Further, our qualitative findings indicated that while dyads enjoyed being able to share the TSW experience, relationship quality was supported rather than changed by group TSW as “bonds” between families and couples had been established over very long periods of time. These findings are confirmed by previous research, suggesting that shared identity, mutuality, and empathic attunement between people with dementia and their family caregivers are built over decades, and this established relationship is most likely to impact continued interconnectedness, partnership, and closeness (Hellström et al., 2007; Wadham et al., 2016). However, it would be interesting to investigate whether music therapy involving group TSW or therapeutic singing sustains relationship quality of people with dementia and their family caregivers over an extended period of time (for example, 52 weeks or more) when compared with usual care in a randomized controlled trial.

There were no statistically significant changes for outcomes measuring depression and quality of life in participants with dementia. However, CSDD ratings demonstrated a clinical change from “probable major depression” to “possible depression” (Alexopoulos et al., 1988), and effect sizes suggested trends toward reduced symptoms of depression and improved mood, behavioral disturbance, and ideational disturbance. To our knowledge, no other previous studies have investigated symptoms of depression and quality of life among people with dementia who participated in TSW. However, a recent systematic review and meta-analysis of seven high-quality randomized controlled trials investigating the effects of active (singing, instrument playing) and receptive (listening) music interventions for people with dementia demonstrated significant reductions in symptoms of depression for programs over 6, 8, and 16 weeks when sessions were facilitated by a qualified music therapist (Li et al., 2019). Our results are consistent with findings from this review, suggesting that symptoms of depression in people with dementia could be sensitive to change in a fully powered randomized controlled trial investigating the effects of group TSW over the short-term.

Conversely, as might be expected with the progression of dementia (Arvanitakis and Bennett, 2019), observed effects from the QoL-AD suggested that both self-rated and proxy-rated quality of life among participants with dementia were reduced from pre to post assessment. However, our results differ from the strong correlation between quality of life and depression among people with dementia reported by the developers of the QoL-AD (Logsdon et al., 2002). Further, our findings are consistent with those of Tamplin et al. (2018), who similarly observed small to medium effects suggesting reduced quality of life for participants with dementia who attended therapeutic group singing with their family caregivers. Effect sizes observed in the current project suggesting decreased quality of life alongside trends toward improved depression could be explained by our qualitative findings. Participants with dementia and their caregivers noted the mentally stimulating aspects of group TSW and described many positive feelings and emotions associated with accomplishment and achievement. Participants also valued the way group TSW stimulated social engagement and connection, factors which correlate with good quality of life and reduced risk of depression (Penninkilampi et al., 2018). However, our qualitative interviews also suggested that some participants with dementia engaged in reflective reminiscence, a finding also indicated in other qualitative research exploring group TSW for people with dementia (Baker and Stretton-Smith, 2017). It is possible that this reminiscence and reflection stimulated insight and an awareness of losses from the past. Thus, symptoms of depression may have been lifted owing to the stimulating social and mental aspects of TSW, but reminiscence and reflection could have made people more aware of loss associated with dementia, and this insight may have impacted how quality of life was perceived.

Our results build on other recent research examining group TSW for family caregivers of people with dementia participating without their family member, which also observed favorable effect sizes for depression (Baker et al., 2018; García-Valverde et al., 2019), as well as self-esteem and anxiety (García-Valverde et al., 2019). While our favorable effect sizes for variables examining mental health align with theory underpinning TSW (Baker, 2015), our results demonstrating observed effects for the physical super-dimension and independent living sub-domain of the AQoL-8D are not as easy to explain. Perhaps small effects suggesting trends toward improved happiness, relationships, depression, and perceptions of caregiver burden, which relate more directly to the aims of TSW, help to explain why family caregivers may have felt more competent and independent about performing the physical and practical aspects of their lives. Nonetheless, relationships between these variables are complex and warrant further investigation to explore how group TSW may have a holistic impact on family caregivers’ quality of life.

Our qualitative findings also help to explain favorable effects for caregivers attending group TSW sessions with their family member and build on previous research examining TSW groups with caregivers alone (Baker et al., 2018). Caregiver participants in our research explained how attending group TSW with their family member was an overwhelmingly positive shared experience. They appreciated the TSW program as an opportunity to participate in music together with their family member and enjoyed observing the achievements and active contributions of their family member and others with dementia in the groups. Family caregivers also described gaining personal satisfaction, insights, and social and emotional support from attending group TSW. However, Baker et al. (2018) recognize that participants in groups designed solely for caregivers were able to speak openly about the full caregiver journey with others in a similar situation, and it is likely that this more in-depth exploration of the caregiver role and experience was curtailed in our groups where family members with dementia were present. These findings suggest that group TSW can be beneficial for family caregivers when they attend with their family member with dementia or on their own depending on whether the aim is to provide opportunities for personal growth, awareness, and coping in the caregiver role or to have meaningful shared experiences with their family member.

While the assessments were generally well tolerated, difficulties with some outcome measures were reported by participants and observed by the assessors. Some family caregivers found that reversed items on the QCPR were confusing, a finding which is consistent with other research with the same population (Tamplin et al., 2018). Further, in keeping with recommendations by the developers of the QoL-AD, some participants with severe dementia (MMSE less than 10) found it difficult to select answers from multiple choices and/or maintain the focus required to complete the measure (Logsdon et al., 2002). Conversely, some family caregivers declined the QoL-AD proxy-outcome because they felt their family member with dementia was able to answer for themselves. Further, some family caregivers who lived separately from their family member with dementia did not feel well enough informed to complete the proxy CSDD. These challenges with collection of self and proxy data led to substantial amounts of missing data for some participants with dementia. Flexible and considered assessment approaches are warranted with this population including careful selection of outcome measures, case by case data collection recognizing a need for either self or proxy assessments as appropriate, and/or involving alternative sources such as formal caregivers if required.

The current study has a number of limitations, which need to be considered alongside the feasibility aims of the study. Observed effects suggested that TSW may have potential to reduce depression in people with dementia and improve quality of life in family caregivers with a fully powered sample. However, this finding should be considered with caution owing to the small sample of only 10 dyads and substantial amounts of missing data for some participants with dementia. The current project also recruited dyads from two diverse contexts, those who were living together in the community and others who lived separately because the person with dementia resided in an aged care home. It is likely that the needs of participants across these two contexts vary considerably. However, subgroup analyses were not appropriate given the small sample size. Regardless of these limitations, results from this feasibility project suggest important benefits from dyad-focused group TSW that can be used to inform further research.

Very few studies have examined dyad-focused group music therapy interventions, and none appears to have investigated group TSW with people with dementia and their family caregivers attending together. Therefore, the current feasibility project provides early evidence which could be used to inform a fully powered trial. Observed effects suggest depression in people with dementia and quality of life in family caregivers might be significantly improved with a fully powered study. Further, secondary outcome measures (QoL-AD and CSDD for people with dementia and the AQoL-8D, PHQ-9, and ZBI for family caregivers) appeared to be sufficiently sensitive to measure change following six weekly group TSW interventions. However, a ceiling effect for the primary outcome measure, the QCPR, along with findings from qualitative interviews, suggests that this outcome measure may not be sensitive to change or relevant in research that attracts dyads who want to participate together. Future research would also benefit from a separate analysis of outcomes for dyads living together in the community and those who live separately because the person with dementia is in residential care. Further, challenges with collection of self and proxy data on outcomes for people with dementia suggest that flexible and considered assessment and data analysis approaches are warranted and need to be factored into research protocols.

In conclusion, qualitative findings helped to explain favorable observed quantitative well-being effects and recognize positive personal and social experiences for individuals, dyads, and groups. In particular, our findings highlighted the value of TSW as a positive social experience, which was mentally and creatively stimulating for both people living with dementia and family caregivers, despite cognitive challenges, musical background or songwriting experience. Future research with a fully powered study informed by results from this feasibility study is recommended to further examine potential psychosocial benefits of dyad-focused group TSW programs attended by people living with dementia and their family caregivers.

## Data Availability Statement

All datasets presented in this study are included in the article/supplementary material.

## Ethics Statement

The studies involving human participants were reviewed and approved by the Education, Fine Arts Music and Business Human Ethics Committee, University of Melbourne. The patients/participants provided their written informed consent to participate in this study. Written, informed consent was obtained from all individual(s) participants OR their legal guardian/next of kin for the publication of any potentially identifiable quotations or data included in this article.

## Author Contributions

IC was responsible for the study design, project management, data analysis, and wrote the majority of the manuscript. PS-S facilitated the intervention, collected quantitative data, conducted qualitative interviews, and contributed to data analysis and writing of the manuscript. JT assisted with conceptualization of the project design, acted as an advisor throughout the project, and contributed to writing of the manuscript. FB and Y-EL provided general support during the project and commented on the final manuscript. All authors contributed to the article and approved the submitted version.

## Conflict of Interest

The authors declare that the research was conducted in the absence of any commercial or financial relationships that could be construed as a potential conflict of interest.

## Footnotes

1 All participant names are pseudonyms; ^∗^ family caregivers.

## References

[B1] Ablitt A. Jones G. V. Muers J. (2009). Living with dementia: a systematic review of the influence of relationship factors. *Aging Ment. Health* 13 497–511. 10.1080/13607860902774436 19629774

[B2] Ahessy B. (2017). Song writing with clients who have dementia: a case study. *Arts Psychother.* 55 23–31. 10.1016/j.aip.2017.03.002

[B3] Alexopoulos G. S. Abrams R. C. Young R. C. Shamoian C. A. (1988). Cornell scale for depression in dementia. *Biol. Psychiatry* 23 271–284. 10.1016/0006-3223(88)90038-8 3337862

[B4] Arvanitakis Z. Bennett D. A. (2019). What is dementia? *Jama* 322:1728. 10.1001/jama.2019.11653 31688886 PMC7455009

[B5] Baker F. A. (2015). *Therapeutic Songwriting: Developments in Theory, Methods, and Practice.* Hampshire: Palgrave Macmillan.

[B6] Baker F. A. Stretton-Smith P. A. (2017). Group therapeutic songwriting and dementia: exploring the perspectives of participants through interpretative phenomenological analysis. *Music Ther. Perspect.* 36 50–66. 10.1093/mtp/mix016

[B7] Baker F. A. Stretton-Smith P. A. Clark I. N. Tamplin J. Lee Y.-E. (2018). A group therapeutic songwriting intervention for family caregivers of people living with dementia: a feasibility study with thematic analysis. *Front. Med. Geriatr. Med.* 5:151. 10.3389/fmed.2018.00151 29872659 PMC5972290

[B8] Baker F. A. Yeates S. (2017). Carers’ experiences of group therapeutic songwriting: an interpretive phenomenological analysis. *Br. J. Music Ther.* 32 8–17. 10.1177/1359457517728914

[B9] Batsch N. L. Mittelman M. S. (2012). *World Alzheimer Report 2012: Overcoming the Stigma of Dementia.* London: Alzheimer’s Disease International.

[B10] Camic P. M. Williams C. M. Meeton F. (2013). Does a ‘Singing Together Group’ improve the quality of life of people with a dementia and their carers? A pilot evaluation study. *Dementia* 12 157–176. 10.1177/1471301211422761 24336767

[B11] Clark I. N. Tamplin J. Baker F. A. (2018). Community-dwelling people living with dementia and their family caregivers’ experiences of therapeutic group singing: a qualitative thematic analysis. *Front. Psychol.* 9:1332. 10.3389/fpsyg.2018.01332 30104994 PMC6077620

[B12] Cohen J. (1988). *Statistical Power Analysis for the Behavioral Sciences*, 2nd Edn New York, NY: Lawrence Erlbaum Associates.

[B13] Cove J. Jacobi N. Donovan H. Orrell M. Stott J. Spector A. (2014). Effectiveness of weekly cognitive stimulation therapy for people with dementia and the additional impact of enhancing cognitive stimulation therapy with a carer training program. *Clin. Interv. Aging* 9 2143–2150. 10.2147/CIA.S66232 25525349 PMC4267515

[B14] Folstein M. F. Folstein S. E. McHugh P. R. (1975). ‘Mini-mental State’: a practical method for grading the cognitive state of patients for the clinician. *J. Psychiatr. Res.* 12 189–198. 1202204 10.1016/0022-3956(75)90026-6

[B15] Fox-Wasylyshyn S. M. El-Masri M. M. (2005). Handling missing data in self-report measures. *Res. Nurs. Health* 28 488–495. 10.1002/nur.20100 16287052

[B16] García-Valverde E. Badia M. Orgaz B. M. Gónzalez-Ingelmo E. (2019). The influence of songwriting on quality of life of family caregivers of people with dementia: an exploratory study. *Nordic J. Music Ther.* 29 4–19. 10.1080/08098131.2019.1630666

[B17] Gorska S. Forsyth K. Maciver D. (2018). Living with dementia: a meta-synthesis of qualitative research on the lived experience. *Gerontologist* 58 e180–e196. 10.1093/geront/gnw195 28069886 PMC5946830

[B18] Greenblat C. (2012). “Dementia caregiving and caregivers,” in *Dementia: A public health Priority*, ed. Bramley D. (Geneva: World Health Organisation), 67–80.

[B19] Groen-van de Ven L. Smits C. Oldewarris K. Span M. Jukema J. Eefsting J. (2017). Decision trajectories in dementia care networks: decisions and related key events. *Res. Aging* 39 1039–1071. 10.1177/0164027516656741 27401681

[B20] Guerchet M. Ali G.-C. Prince M. Wu Y.-T. (2016). “The incidence of dementia,” in *Proceedings of the World Alzheimer Report 2015: The Global Impact of Dementia: An Analysis of Prevalence, Incidence, Cost and Trends* (London: Alzheimer’s Disease International).

[B21] Hebert R. Bravo G. Preville M. (2000). Reliability, validity and reference values of the Zarit Burden Interview for assessing informal caregivers of community-dwelling older persons with dementia. *Can. J. Aging* 19 494–507. 10.1017/S0714980800012484

[B22] Hellström I. Nolan M. Lundh U. (2005). We do things together”: a case study of “couplehood” in dementia. *Dementia* 4 7–22. 10.1177/1471301205049188

[B23] Hellström I. Nolan M. Lundh U. (2007). Sustaining ‘couplehood’: spouses’strategies for living positively with dementia. *Dementia* 6 383–409. 10.1177/1471301207081571

[B24] Hong I. S. Choi M. J. (2011). Songwriting orientated activities improve the cognitive functions of the aged with dementia. *Arts Psychother.* 38 221–228. 10.1016/j.aip.2011.07.002

[B25] Kitwood T. Bredin K. (1992). Towards a theory of dementia care: personhood and well-being. *Ageing Soc.* 12 269–287. 10.1017/s0144686x0000502x 11654434

[B26] Kroenke K. Spitzer R. L. William J. B. (2001). The PHQ-9: validity of a brief depression severity measure. *J. Gen. Intern. Med.* 16 606–613. 10.1046/j.1525-1497.2001.016009606.x 11556941 PMC1495268

[B27] Li H. C. Wang H. H. Lu C. Y. Chen T. B. Lin Y. H. Lee I. (2019). The effect of music therapy on reducing depression in people with dementia: a systematic review and meta-analysis. *Geriatr. Nurs.* 40 510–516. 10.1016/j.gerinurse.2019.03.017 31056209

[B28] Logsdon R. G. Gibbons L. E. McCurry S. M. Teri L. (1999). Quality of life in Alzheimer’s Disease: pateint and caregiver reports. *J. Ment. Health Aging* 5 21–32.

[B29] Logsdon R. G. Gibbons L. E. McCurry S. M. Teri L. (2002). Assessing quality of life in older adults with cognitive impairment. *Psychosom. Med.* 64 510–519. 10.1097/00006842-200205000-00016 12021425

[B30] Maxwell A. Ozmen M. Iezzi A. Richardson J. (2016). Deriving population norms for the AQoL-6D and AQoL-8D multi-attribute utility instruments from web-based data. *Qual. Life Res.* 25 3209–3219. 10.1007/s11136-016-1337-z 27344318

[B31] McDermott O. Crellin N. Ridder H. M. Orrell M. (2013). Music therapy in dementia: a narrative synthesis systematic review. *Int. J. Geriatr. Psychiatry* 28 781–794. 10.1002/gps.3895 23080214

[B32] McDermott O. Orrell M. Ridder H. M. O. (2014). The importance of music for people with dementia: the perspectives of people with dementia, family carers, staff and music therapists. *Ageing Ment. Health* 18 706–716. 10.1080/13607863.2013.875124 24410398 PMC4066923

[B33] Moniz-Cook E. Vernooij-Dassen M. Woods R. Verhey F. Chattat R. De Vugt M. (2008). A European consensus on outcome measures for psychosocial intervention research in dementia care. *Aging Ment. Health* 12 14–29. 10.1080/13607860801919850 18297476

[B34] Osman S. E. Tischler V. Schneider J. (2016). ‘Singing for the Brain’: a qualitative study exploring the health and well-being benefits of singing for people with dementia and their carers. *Dementia* 15 1326–1339. 10.1177/1471301214556291 25425445 PMC5089222

[B35] Penninkilampi R. Casey A. N. Singh M. F. Brodaty H. (2018). The association between social engagement, loneliness, and risk of dementia: a systematic review and meta-analysis. *J. Alzheimers Dis.* 66 1619–1633. 10.3233/JAD-180439 30452410

[B36] Rausch A. Caljouw M. A. van der Ploeg E. S. (2017). Keeping the person with dementia and the informal caregiver together: a systematic review of psychosocial interventions. *Int. Psychogeriatr.* 29 583–593. 10.1017/S1041610216002106 27890029

[B37] Richardson J. Iezzi A. Khan M. A. Maxwell A. (2014a). Validity and reliability of the Assessment of Quality of Life (AQoL)-8D multi-attribute utility instrument. *Patient* 7 85–96. 10.1007/s40271-013-0036-x 24271592 PMC3929769

[B38] Richardson J. Sinha K. Iezzi A. Khan M. A. (2014b). Modelling utility weights for the Assessment of Quality of Life (AQoL)-8D. *Qual. Life Res.* 23 2395–2404. 10.1007/s11136-014-0686-8 24719017

[B39] Seng B. K. Luo N. Ng W. Y. Lim J. Chionh H. L. Goh J. (2010). Validity and reliability of the Zarit Burden Interview in assessing caregiving burden. *Ann. Acad. Med. Singapore* 39 758–763. 21063635

[B40] Silber F. Hes J. (1995). The use of songwriting with patients diagnosed with Alzheimer’s disease. *Music Ther. Perspect.* 13 31–34. 10.1093/mtp/13.1.31

[B41] Smith J. A. Flowers P. Larkin M. (2009). *Interpretative Phenomenological Analysis: Theory, Method and Research.* London: Sage.

[B42] Spruytte N. Van Audenhove C. Lammertyn F. Storms G. (2002). The quality of the caregiving relationship in informal care for older adults with dementia and chronic psychiatric patients. *Psychol. Psychother. Theory Res. Pract.* 75 295–311. 10.1348/147608302320365208 12396755

[B43] Tamplin J. Clark I. N. (2019). “Therapeutic music interventions to support people with dementia living at home with their family caregivers,” in *Music and Dementia: From Cognition to Therapy*, eds Baird A. Garrido S. Tamplin J. (New York, NY: Oxford University Press), 269–287. 10.1093/oso/9780190075934.003.0013

[B44] Tamplin J. Clark I. N. Lee Y. C. Baker F. A. (2018). Remini-Sing: a feasibility study of therapeutic group singing to support relationship quality and wellbeing for community-dwelling people living with dementia and their family caregivers. *Front. Med.* 5:245. 10.3389/fmed.2018.00245 30234118 PMC6127293

[B45] Unadkat S. Camic P. M. Vella-Burrows T. (2017). Understanding the experience of group singing for couples where one partner has a diagnosis of Dementia. *Gerontologist* 57 469–478. 10.1093/geront/gnv698 26783138

[B46] Van Bruggen S. Gussekloo J. Bode C. Touwen D. P. Engberts D. P. Blom J. W. (2016). Problems experienced by informal caregivers with older care recipients with and without cognitive impairment. *Home Health Care Serv. Q.* 35 11–24. 10.1080/01621424.2016.1145166 27018745 PMC4917916

[B47] Wadham O. Simpson J. Rust J. Murray C. (2016). Couples’ shared experiences of dementia: a meta-synthesis of the impact upon relationships and couplehood. *Aging Ment. Health* 20 463–473. 10.1080/13607863.2015.1023769 25811103

[B48] WHO (2017). *Global Action Plan on the Public Health Response to Dementia 2017–2025.* Geneva: WHO.

[B49] Wongpakaran N. Wongpakaran T. van Reekum R. (2013). Discrepancies in cornell scale for depression in Dementia (CSDD) items between residents and caregivers, and the CSDD’s factor structure. *Clin. Interv. Aging* 8 641–648. 10.2147/CIA.S45201 23766640 PMC3677808

[B50] Yalom I. Leszcz M. (2005). *Theory and Practice of Groyp Psychotherapy*, 5th Edn New York, NY: Basic Books.

[B51] Zarit S. H. Reever K. E. Bach-Peterson J. (1980). Relatives of the impaired elderly: correlates of feelings of burden. *Gerontologist* 20 649–655. 10.1093/geront/20.6.649 7203086

